# Oral Hygiene Instructions With Plaque-Disclosing Agents to Improve Self-Performed Dental Plaque Control: A Case Report

**DOI:** 10.7759/cureus.72205

**Published:** 2024-10-23

**Authors:** Shilpa Kamra, Karthikeyan Ramalingam, Salaria SK, Manish Sukhija, Sandeep Sihmar

**Affiliations:** 1 Periodontology, Shree Guru Gobind Singh Tricentenary (SGT) University, Gurugram, IND; 2 Oral Pathology and Microbiology, Saveetha Dental College and Hospitals, Saveetha Institute of Medical and Technical Sciences, Saveetha University, Chennai, IND; 3 Periodontology and Implantology, Misha's Dental Clinic and Implant Centre, Meerut, IND; 4 Periodontology, Surendera Dental College and Research Institute, Sri Ganganagar, IND; 5 Oral Pathology, Daswani Dental College and Research Centre, Kota, IND

**Keywords:** bleeding gums, dental plaque, dentifrice, gingival index, gingivitis, oral hygiene, plaque control, plaque index, tooth brushing, toothpaste

## Abstract

Professional application of plaque-disclosing solutions (PDS) by dental surgeons can promote patient awareness. Verbal communication of oral hygiene instructions and demonstration of brushing techniques can improve self-performed dental plaque control. Dental plaque is a major cause of many oral diseases, and prompt removal ensures better oral health. In this case report, we demonstrate the usage of such a technique for improving the oral hygiene of an adolescent. Commercially available PDS was used at baseline to document the Turesky-modified Quigley-Hein plaque index and Loe and Silness gingival index scores. Oral hygiene measures and proper brushing techniques were demonstrated. The patient was followed up after 12 hours, 24 hours, one week, and four weeks to monitor the variations in plaque and gingival index. We observed a progressive reduction in plaque and gingival index, indicating an efficient self-performed dental plaque control.

## Introduction

Dental plaque (DP) accumulates on all hard and soft surfaces within the oral cavity, including fixed and removable prostheses. If plaque control is inadequate, it can initially cause gingival inflammation, which, if left untreated, may extend into the periodontal tissues. This progression can result in the gradual loss of attachment structures, including the alveolar bone [1], leading to tooth mobility. Additionally, localized inflammation may adversely affect the body's immune response and health [2]. DP formation and maturation happen over a few weeks. Plaque control is essential to treat and prevent dental caries and periodontal diseases [1,2]. Mechanical plaque removal using a toothbrush and toothpaste is commonly practiced, with flossing also recommended for controlling DP [3,4]. Toothbrushing removes DP from the tooth surface, and the gingiva is massaged to induce keratinization [4]. Preventive dentistry acknowledges various brushing techniques such as horizontal scrub, Fones, rolling method, Bass, Charters, Stillman, and others [4]. However, the dental community acknowledges that self-performed mechanical plaque control often falls short of optimal oral hygiene. Users may not brush frequently enough for the recommended duration or may struggle to master proper brushing techniques.

Oral hygiene status is determined by the identification of DP by the naked eye or by applying disclosing agents. DP indices can be non-quantitative visualization or quantitative evaluation of plaque deposits [4]. Disclosing solutions can contain stains such as iodine, methylene blue, and basic Fuchsin that bind to the plaque and allow easy visualization. It helps in the objective estimation of DP. It also ensures patient motivation, raises patient awareness, and improves self-performed plaque control [4-6]. Our case report deals with the progress of self-performed plaque control in an adolescent male using a plaque-disclosing solution (PDS), demonstration, and reinforcement of proper brushing techniques.

## Case presentation

A 17-year-old adolescent male reported to the outpatient department of Surendera Dental College and Research Institute for a dental checkup. His past medical and surgical history were non-contributory. He brushes once a day with a hard toothbrush and tooth powder. He has not undergone any dental procedures to date. Informed consent was obtained from the parent and the patient for further examination and treatment.

The extraoral examination did not reveal any abnormalities. The intraoral examination revealed foci of DP with gingival redness in multiple quadrants. Calculus formation was not evident, and there was no evidence of dental caries. Gingival probing was tender in the anterior quadrants, and multiple foci of bleeding upon probing were also evident. There was no periodontal pocket formation. A diagnosis of chronic localized gingivitis was made.

**Figure 1:**
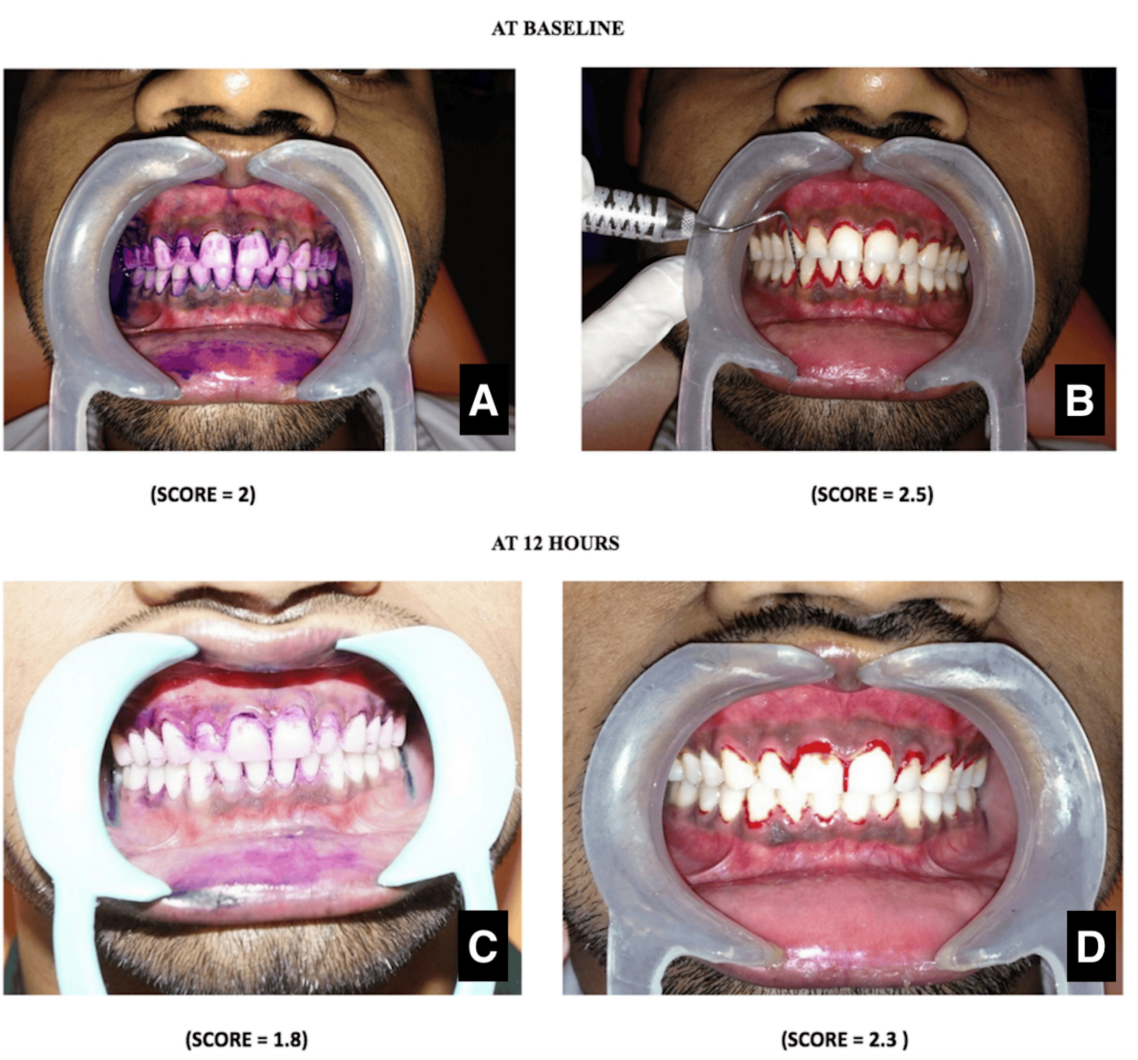
Clinical images The Turesky-modified Quigley-Hein plaque index score at baseline (A) and after 12 hours (C); the Loe and Silness gingival index score at baseline (B) and after 12 hours (D).

The treatment plan was to give oral hygiene instructions and regular follow-up. Per manufacturer specifications, the patient was asked to perform an oral rinse using a two-tone PDS (DPI Alphaplac, Jalandhar, India). The old plaque was colored blue, and the new plaque was colored pink. The clinical pictures were photographed at baseline. The Turesky modification of the Quigley-Hein plaque index score and Loe and Silness gingival index score were recorded. The plaque index is scored from 0 to 5 based on the plaque present on the tooth surface. No plaque is scored as 0, and more than two-thirds of the tooth surface covered with plaque is scored as 5. The gingival index is scored from 0 to 3. Bleeding from the gingiva is recorded as 2. The plaque index score at the baseline was 2, while the gingival index score at the baseline was 2.5 (Figure 1).

The patient was instructed to use a soft-bristled toothbrush and toothpaste containing triclosan and sodium monofluorophosphate. The patient was instructed and demonstrated brushing techniques using the rolling and Bass methods. The patient was asked to perform the demonstrated brushing technique. The patient's brushing pattern was observed, and the dental surgeon guided relevant modifications.

The patient was asked to report back after 12 hours. A PDS was used, and clinical photographs were taken. The plaque index score was 1.8, and the gingival index score was 2.3. The patient was asked to perform the new brushing technique. The patient's brushing pattern was observed, and relevant modifications were suggested again (Figure 1).

**Figure 2:**
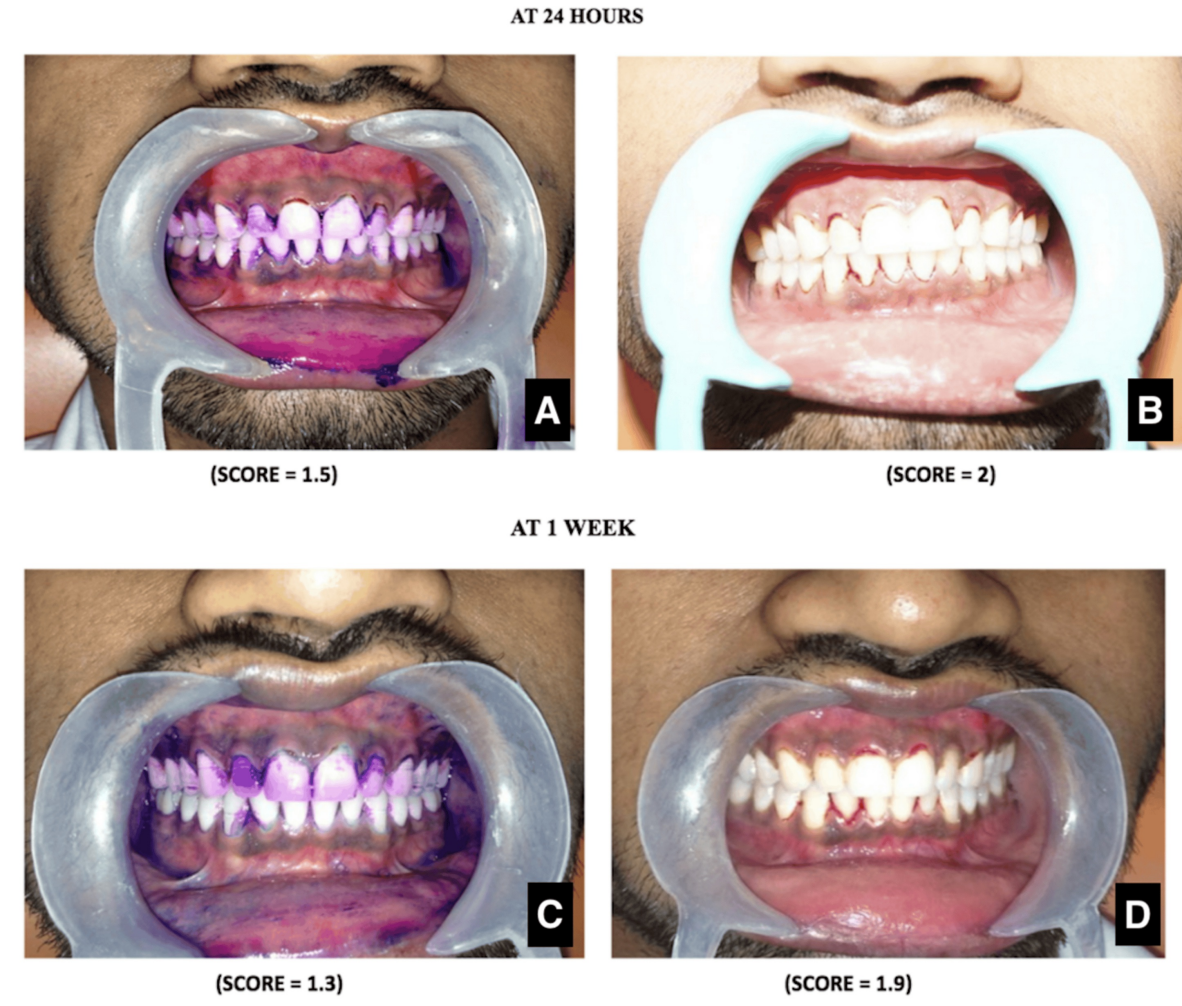
Clinical images The Turesky-modified Quigley-Hein plaque index score after 24 hours (A) and after one week (C); the Loe and Silness gingival index score after 24 hours (B) and after one week (D).

The patient was asked to report back after 24 hours. A PDS was used, and clinical photographs were taken. The plaque index score was 1.5, and the gingival index score was 2. The patient was asked to perform the new brushing technique. The patient's brushing pattern was observed, and relevant modifications were suggested again to reinforce the new brushing method (Figure 2).

The patient was instructed to report after one week. A PDS was used, and clinical photographs were taken. The plaque index score was 1.3, and the gingival index score was 1.9. The patient was asked to perform the brushing technique. The patient's brushing pattern was observed, and relevant modifications were suggested again to reinforce the new brushing method. The patient was motivated by the progress in his oral hygiene (Figure 2).

**Figure 3:**
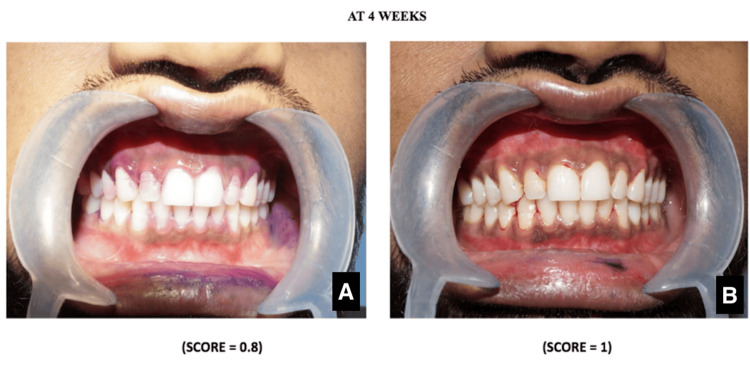
Clinical images The Turesky-modified Quigley-Hein plaque index score after four weeks (A); the Loe and Silness gingival index score after four weeks (B).

The patient was instructed to report after four weeks. A PDS was used, and clinical photographs were taken. The plaque index score was 0.8, and the gingival index score was 1 (Figure 3). He was commended for the continuous improvement of oral hygiene maintenance. The patient was asked to perform the brushing technique. The patient's brushing pattern was observed, and relevant modifications were suggested again to reinforce the new brushing method. The patient was motivated and instructed to continue with the same process.

The patient had moved from the city, and a voice call after six months verified that he continues to brush twice daily with a soft toothbrush and fluoridated toothpaste, employing the instructed toothbrushing technique.

## Discussion

It is widely acknowledged that DP accumulation is a continuous process and serves as the primary factor triggering the host's immune response. The interaction between the host's immune system and the plaque microbiota is influenced by various environmental, genetic, and systemic factors. It can accelerate periodontal tissue destruction and contribute to systemic health conditions, and vice versa [7]. Prevention of plaque-induced periodontal inflammation requires regular home oral care practices such as tooth brushing, the use of dentifrices, and flossing, which are commonly recommended. However, despite these efforts, many individuals still struggle to achieve the level of oral hygiene necessary for maintaining periodontal health [8]. This challenge has led researchers to explore the potential of enhancing oral hygiene by incorporating specific chemotherapeutic agents into toothpaste formulations. For instance, the Colgate Total toothpaste contains 0.3% triclosan, 2% copolymer, and 0.243% sodium fluoride. It is clinically proven and endorsed by the Food and Drug Administration for the prevention of plaque and gingivitis. It is also recognized by the American Dental Association [9,10].

Regular but inefficient brushing leads to a high prevalence of gingivitis and could be due to a lack of compliance among adolescents [11]. Peng et al. [6] reported higher gingival and plaque indices among teenagers and females. Novel agents derived from natural substances [12] can improve oral health and also avoid the progression of gingival disease into advanced periodontal disease [13,14]. Plaque indices can help assess the cleansing efficacy of a technique or a product [4]. Jones and Morrison [15] have recommended the usage of the plaque index and gingival index for oral health evaluation in patients with Down's syndrome. Kumaraguru et al. [16] have systematically analyzed audio-tactile measures with oral health education among visually impaired adolescents.

Boneta et al. [9] and Mankodi et al. [10] have significantly demonstrated that triclosan-containing dentifrice reduces supragingival plaque and gingivitis. Yaacob et al. [17] have reported a key finding that powered toothbrushes are more efficient in reducing plaque and gingivitis than manual brushing. A comprehensive analysis of clinical parameters with patient-centric outcomes is important to improve the patient's overall quality of life (QoL) [18].

We have used a PDS to monitor the plaque levels and motivate the patient. We also assessed the plaque index and gingival index scores over four weeks to evaluate the effectiveness of self-performed DP control by proper tooth brushing technique, using a soft toothbrush, and using toothpaste with triclosan and fluoride. Our case report shows the patient's observed improvement in overall plaque control.

## Conclusions

Clinicians are encouraged to incorporate plaque-disclosing techniques into routine dental practice for the enhancement of patient awareness and to motivate individuals to maintain optimal oral hygiene. By visualizing plaque accumulation, patients gain a better understanding of their oral hygiene status, thereby increasing compliance with recommended oral care practices. Regular follow-up appointments are critical in ensuring that patients adhere to oral hygiene protocols and allow for the early detection and intervention of plaque-induced oral diseases, such as gingivitis and periodontitis. This proactive approach can significantly reduce the progression of periodontal disease and mitigate associated systemic health risks. The present case report underscores the importance of these strategies by demonstrating their effectiveness in improving oral health outcomes. This case report is a valuable contribution supporting the implementation of plaque-disclosing methods as a standard component of preventive dental care.

## References

[1] Peeran SW, Ramalingam K (2021). Essentials of Periodontics and Oral Implantology. JPS Publication, Chennai, India.

[2] D'Elia G, Floris W, Marini L, Corridore D, Rojas MA, Ottolenghi L, Pilloni A (2023). Methods for evaluating the effectiveness of home oral hygiene measures—a narrative review of dental biofilm indices. Dent J (Basel).

[3] Duane B, Yap T, Neelakantan P (2023). Mouthwashes: alternatives and future directions. Int Dent J.

[4] Bok HJ, Lee CH (2020). Proper tooth-brushing technique according to patient's age and oral status. Int J Clin Prev Dent.

[5] Oliveira LM, Pazinatto J, Zanatta FB (2021). Are oral hygiene instructions with aid of plaque-disclosing methods effective in improving self-performed dental plaque control? A systematic review of randomized controlled trials. Int J Dent Hyg.

[6] Peng Y, Wu R, Qu W (2014). Effect of visual method vs plaque disclosure in enhancing oral hygiene in adolescents and young adults: a single-blind randomized controlled trial. Am J Orthod Dentofacial Orthop.

[7] Badiger AB, Gowda TM, Chandra K, Mehta DS (2019). Bilateral interrelationship of diabetes and periodontium. Curr Diabetes Rev.

[8] Bui FQ, Almeida-da-Silva CL, Huynh B (2019). Association between periodontal pathogens and systemic disease. Biomed J.

[9] Boneta AE, Aguilar MM, Romeu FL, Stewart B, DeVizio W, Proskin HM (2010). Comparative investigation of the efficacy of triclosan/copolymer/sodium fluoride and stannous fluoride/sodium hexametaphosphate/zinc lactate dentifrices for the control of established supragingival plaque and gingivitis in a six-month clinical study. J Clin Dent.

[10] Mankodi S, Chaknis P, Panagakos FS, DeVizio W, Proskin HM (2011). Comparative investigation of a dentifrice containing triclosan/copolymer/sodium fluoride and specially-designed silica and a dentifrice containing 0.243% sodium fluoride in a silica base for the control of established supra-gingival plaque and gingivitis: a 6-month clinical study. Am J Dent.

[11] Eidenhardt Z, Ritsert A, Shankar-Subramanian S, Ebel S, Margraf-Stiksrud J, Deinzer R (2021). Tooth brushing performance in adolescents as compared to the best-practice demonstrated in group prophylaxis programs: an observational study. BMC Oral Health.

[12] M A, I MA, Ramalingam K, Shanmugam R (2024). Biomedical applications of lauric acid: a narrative review. Cureus.

[13] Peeran SW, Ramalingam K, Sethuraman S, Thiruneervannan M (2024). Furcation involvement in periodontal disease: a narrative review. Cureus.

[14] Alam MN, Ibraheem W, Ramalingam K, Sethuraman S, Basheer SN, Peeran SW (2024). Identification, evaluation, and correction of supracrestal tissue attachment (previously biologic width) violation: a case presentation with literature review. Cureus.

[15] Jones D, Morrison J (2016). Preventative therapies and periodontal interventions for Down syndrome patients. Evid Based Dent.

[16] Kumaraguru M, D S, Yuwanati M, I MA (2024). Effectiveness of audio-tactile performance versus other oral health education methods in improving oral health in visually impaired children and adolescents: a systematic review. Cureus.

[17] Yaacob M, Worthington HV, Deacon SA, Deery C, Walmsley AD, Robinson PG, Glenny AM (2014). Powered versus manual toothbrushing for oral health. Cochrane Database Syst Rev.

[18] Sindhusha VB, Rajasekar A (2024). Assessment of clinical and patient-centered outcomes in nonsurgical periodontal therapy. Cureus.

